# An electrochemical aptasensor for detection of *Helicobacter pylori* based on AuNPs and AgNPs-GO nanoparticles

**DOI:** 10.3389/fbioe.2025.1619336

**Published:** 2025-07-09

**Authors:** Xiaojuan You, Mingyi Shao, Huadong Wang, Rui Zhu, Xinwei Liu, Lei Dong, Yuesheng Gong, Yongwei Li

**Affiliations:** ^1^ Central Laboratory, Zhengzhou Anorectal Hospital, Zhengzhou, China; ^2^ The First Affiliated Hospital, Henan University of Chinese Medicine, Zhengzhou, China; ^3^ The Second Clinical Medical College, Henan University of Chinese Medicine, Zhengzhou, China; ^4^ Longhu Laboratory, Zhengzhou, China; ^5^ Medical Device Review Division I, Henan Center For Drug Evaluation and Inspection, Zhengzhou, China

**Keywords:** *Helicobacter pylori*, AgNPs-GO, AuNPs, streptavidin, aptasensor

## Abstract

Helicobacter pylori is one of the main causes of gastritis and gastric ulcer. Early detection of *H. pylori* is of great significance for the prevention of gastric cancer. Herein, a sensitive electrochemical aptasensor using AgNPs-GO as redox probes was established for the specific detection of *H. pylori* in blood serum and stool samples. Firstly, AgNPs-GO nanocomposites were obtained by *in-situ* reduction of AgNPs on graphene oxide (GO) surface with glucose as reducing agent, in which AgNPs showed good biocompatibility and chemical stability, as well as redox property, and GO provided a large surface area to assemble a large number of AgNPs. Subsequently, the electrodeposition of AuNPs further improved the conductivity of the aptasensor. Additionally, the streptavidin was introduced into the aptasensor to effectively bind the biotin-modified aptamers. In this way, aptamers could be tethered to the surface through SA-biotin linkage, and *H. pylori* was selectivly binded by the aptamers subsequently. Under optimal conditions, the aptasensor could detect *H. pylori* in a wide concentration range (10^1^ CFU mL^−1^–10^8^ CFU mL^−1^) with a low detection limit of 3 CFU mL^−1^. What’s more, the developed method showed excellent performance in practical application, which provided a promising possibility for the detection of other pathogens in clinical diagnosis.

## 1 Introduction

*Helicobacter pylori*, a kind of gram-negative bacterium, has a wide range of pathogenic, which can cause gastritis, gastric ulcer, gastric cancer and other diseases (Usui et al., 2023; Elbehiry et al., 2023; Zhang et al., 2022). It is classified as a Class I carcinogen by the World Health Organization (Xu et al., 2024). More than half of the world’s population is suffering the chronical infection of *H. pylori* (Tran et al., 2024). Therefore, rapid and accurate detection of *H. pylori* is of great significance for monitoring and preventing *H. pylori* infection related diseases.

Clinical detection of *H. pylori* can be divided into invasive and non-invasive methods. Invasive methods mainly include endoscopy, histopathology, rapid urease test, bacterial isolation and culture. While non-invasive methods mainly include urea breath test, fecal antigen detection and serum antibody detection (Spagnuolo et al., 2024). Because of the invasive operation and time-consuming process, non-invasive detection methods are commonly used in clinical practice (Bordin et al., 2021). Although urea breath test was viewed as the gold standard for diagnosing of *H. pylori* infection, the results of urea breath test may be affected by oral colonization of *H. pylori* and lower carbon dioxide production in children than in adults, reducing the specificity of the test (Said and El-Nasser, 2024). *H. pylori* antibodies can persist in circulation system at least 6 months after eradication therapy of *H. pylori*, leading to false positives and inappropriate treatment using serum antibody test (Al Ofairi et al., 2024). *H. pylori* stool antigen detection method has the advantages of rapid and simple (Ren et al., 2023), but when the antigen concentration in the sample is low, it is easy to appear false negative results. Therefore, we still need to establish a new method for detecting *H. pylori*, which can not only play the advantages of convenient sampling, but also have high sensitivity, selectivity and low detection cost, so as to provide more choices for clinical diagnosis and epidemiological investigation.

Aptamers, like antibodies, have a strong desire for their targets (Negahdary, 2020). Furthermore, aptamers are superior in chemical modification, stability, and finesse in the fabrication of nanostructured devices than antibodies (Abedi et al., 2024). The aptamer-based electrochemical sensor has the ascendancy of simple operation, high sensitivity, fast response speed, low instrument cost and easy miniaturization (Rahimizadeh et al., 2023). It is widely used in medical diagnosis, environmental monitoring, food safety detection and other field (Wang J. et al., 2024; Cossettini et al., 2024). Advances in nanotechnology and the use of advanced nanomaterials have greatly improved the detection performance of electrochemical biosensors. Graphene has been widely used in electrochemical biosensors because of its excellent properties such as high conductivity, large specific surface area and good biocompatibility. However, the easily agglomeration of graphene in aqueous solutions limits its application (Di Matteo et al., 2024). Graphene oxide (GO), one of the derivatives of graphene, contains rich oxygen-containing groups on its surface and edge, making it a certain hydrophilic performance and can be used as an excellent carrier for loading various nanoparticles due to the large specific area (Li et al., 2024). In recent years, gold nanoparticles (AuNPs) and silver nanoparticles (AgNPs) are often used in biosensors to realize sensitive detection of targets because of their high conductivity, good biocompatibility and strong adsorption ability (Yu et al., 2024; Phonklam et al., 2024; Wang R. et al., 2024; Wei et al., 2023). In addition, AgNPs also have redox activity, which avoid the application of additional redox probes (Pan et al., 2024). Streptavidin (SA) is a homologous tetramer protein secreted by *Streptomyces avidinii* and has a high affinity with biotin. The tight and specific binding between SA and biotin is fast and can withstand the influence of pH, temperature and organic solvents (Chen et al., 2023). One molecule of SA can bind four biotin molecules with high affinity and selectivity, leading to the amplify of weak signals and improving the detection sensitivity of low-abundance targets (Liu et al., 2023).

In this study, we aimed to design an electrochemical aptasensor for sensitive detection of *H. pylori*. To achieve maximum sensitivity, we employed a variety of strategies. On the one hand, by combining GO and AgNPs, the nanocomposites could exhibit large surface area, good water solubility, biocompatibility and electrical conductivity, as well as redox activity. On the other hand, GO with a larger specific surface area could be loaded with more AgNPs, meaning that the sensor may show higher sensitivity. Secondly, the excellent biocompatibility and electrical conductivity of AuNPs could enhance the electrical conductivity of the electrode and the loading capacity of SA. SA was used to effectively bind with biotin modified aptamer, thus further improving the sensitivity of the detection method. To the best of our knowledge, there were no prior reports on preparation of aptasensor based on SA and AuNPs/AgNPs-GO for detection of *H. pylori.* Importantly, the established aptasensor could be used for the detection of *H. pylori* in blood serum and stool samples with high accuracy. This new biosensor system was expected to be a potential tool for trace level analysis of pathogenic organisms.

## 2 Materials and methods

### 2.1 Reagents and materials

GO was purchased from Jiangsu XFNANO Technology Co., LTD. (Nanjing, China). Streptavidin, bovine serum albumin (BSA), gold chloride trihydrate (HAuCl_4·_·3H_2_O), silver nitrate and ammonia were purchased from Shanghai Aladdin Biochemical Technology Co., LTD. (Shanghai, China). Nafion was purchased from Shanghai Macklin Biochemical Technology Co., LTD. (Shanghai, China). Biotin-modified *H. pylori* aptamers were synthesized by Sangon Biotech (Shanghai) Co., LTD. (Shanghai, China) with the following sequence: 5′-AGT​GTG​CTC​TTC​TCA​GGT​CTC​GGC​GCG​GTT​GTG​GGT​ACC​TAG​GGT​TGT​TGT​TGC​TTC​TCA​GCA​GTG​TCT​CAG​CAT​ACG​CA-3' (Roushani et al., 2022) biotin, the specific synthesis methods of biotin-modified *H. pylori* aptamers were detailed in the Supplementary Material. All reagents were prepared in ultrapure deionized water.

### 2.2 Instruments

All electrochemical tests were completed by the CHI660E Electrochemical workstation (Shanghai Chenhua Instrument Co., LTD., China) with a three-electrode system including a modified gold working electrode (d = 4 mm), a platinum counter electrode and a saturated calomel reference electrode (SCE). Transmission electron microscope (TEM, JEM-1400, Japan Electronics Co., Ltd., Japan), scanning electron microscope (SEM, auriga-bu, zeiss, Germany), X-ray diffractometer (Ultima IV, Japan) and ultraviolet-visible spectrophotometer (Nanodrop 2000c; Thermo Fisher Scientific Co., Ltd., United States) were used to analyze the morphology and composition of the nanocomposites.

### 2.3 Synthesis of AgNPs-GO nanocomposites

The AgNPs-GO nanocomposites were synthesized according to a previously reported method with minor changes (Wu et al., 2013). In short, 0.5 g of glucose was added to 10 mL of GO solution under magnetic agitation. At the same time, 0.55 M of NH_3_·H_2_O was slowly added to 6.7 mL of AgNO_3_ solution until the precipitation suddenly disappeared, then the solution was slowly added to the GO solution. After a stir of 3 min, the solution was stood at room temperature for 1.5 h. Then, the final solution was centrifuged for 10 min to discard the supernatant and washed with deionized water for 3 times. In order to enhance the dispersion of AgNPs-GO nanocomposites in solution, AgNPs-GO nanocomposites were dissolved in 0.4% Nafion ethanol solution for 1 h by ultrasonic treatment.

### 2.4 Fabrication of the electrochemical aptasensor

**FIGURE 1:**
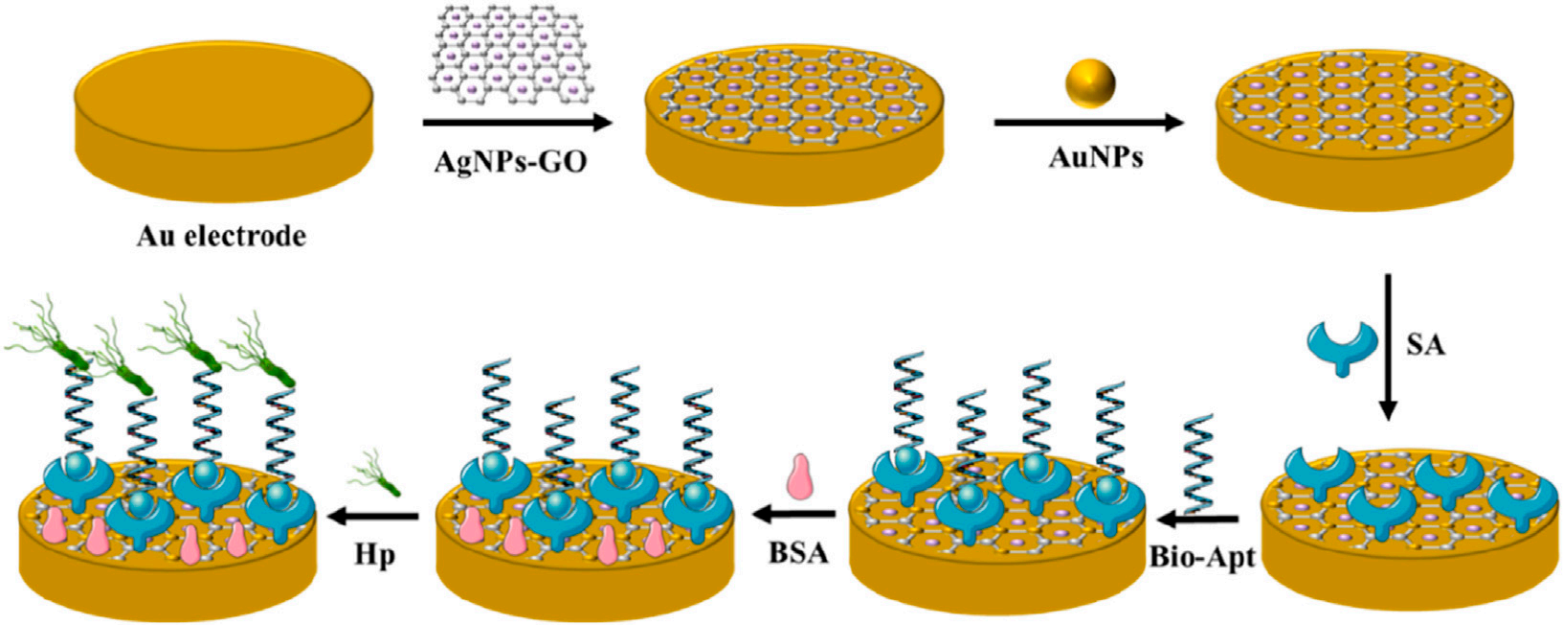
Schematic representation of the proposed *Helicobacter pylori* aptasensor.

The preparation process of the electrochemical aptasensor was shown in Figure 1. Firstly, the gold electrode was polished with 0.05 μm alumina and washed with deionized water to remove physically adsorbent impurities. Next, AgNPs-GO solution (10 μL, 1.0 mg mL^−1^) was coated to the gold electrode and dried at room temperature. In order to further improving the conductivity of the electrode, the modified electrode was placed in HAuCl_4_ solution (1%) for potentiostatic electrodeposition for 25s at −0.2 V to obtain AuNPs/AgNPs-GO modified gold electrode. Then, streptavidin (20 μL, 1.25 mg mL^−1^) was added to the modified electrode and incubated at 37°C for 40 min. Streptavidin could firmly fix on the surface of the modified electrode by Au-N bond. Then the biotin-modified aptamer (20 μL, 2 μM) was added to the surface of the modified electrode and incubated at 4°C for 16 h. The biotin-modified aptamer was fixed to the surface of the modified electrode through the specific adsorption of streptavidin and biotin. Then 20 μL BSA (0.25%) was dripped onto the electrode surface and incubated for 40 min to prevent non-specific adsorption on AuNPs surface. After each step of modification, the modified electrode needed to be cleaned with double distilled water to eliminate physical adsorption. For quantitative analysis, 20 μL of *H. pylori* solution with different concentrations were added to the modified electrode and incubated for 40 min. After specific recognition reaction between *H. pylori* and aptamer, a *H. pylori*-aptamer complex was formed on the electrode surface, which further hindered the electron transfer and thus reduced electrochemical signals. Therefore, the quantitative detection of *H. pylori* could be achieved by tracking this electrochemical signal. All electrochemical measurements were performed at room temperature.

### 2.5 Experimental measurements

The DPV from −0.2–0.3 V with the modulation amplitude of 0.05 V, pulse width of 0.05s and sample width of 0.0167s, was carried out in 5 mL PBS (0.1 M, pH 7.0) to record the electrochemical signals for quantitative detection of *H. pylori.* The assembly process of the modified electrode was characterized by CV measurements with the scanning from −0.3–0.4 V at the scan rate of 50 mV/s in 5 mL PBS (0.1 M, pH 7.0) and EIS measurements with init E of 0.226 V in PBS (0.1 M, pH 7.0) containing 5.0 mM [Fe(CN)6]^3-/4-^.

### 2.6 Preparation of the bacteria

*Staphylococcus aureus*, *Klebsiella pneumoniae*, *Salmonella enterica* and *Acinetobacter baumannii*, which were isolated in our laboratory, were used to investigate the selectivity of the prepared aptasensor. All the bacteria were cultured in LB medium. *H. pylori* (ATCC43504) was cultured on Columbia blood plate medium. All strains were diluted with PBS (0.1 M, pH 7.4). The concentration of the bacteria was adjusted to 0.5 McFarland turbidity. *H. pylori* with different concentrations from 10^1^ to 10^8^ CFU mL^−1^ were used for aptasensor detection.

## 3 Results and discussion

### 3.1 Characterization of nanocomposites

**FIGURE 2:**
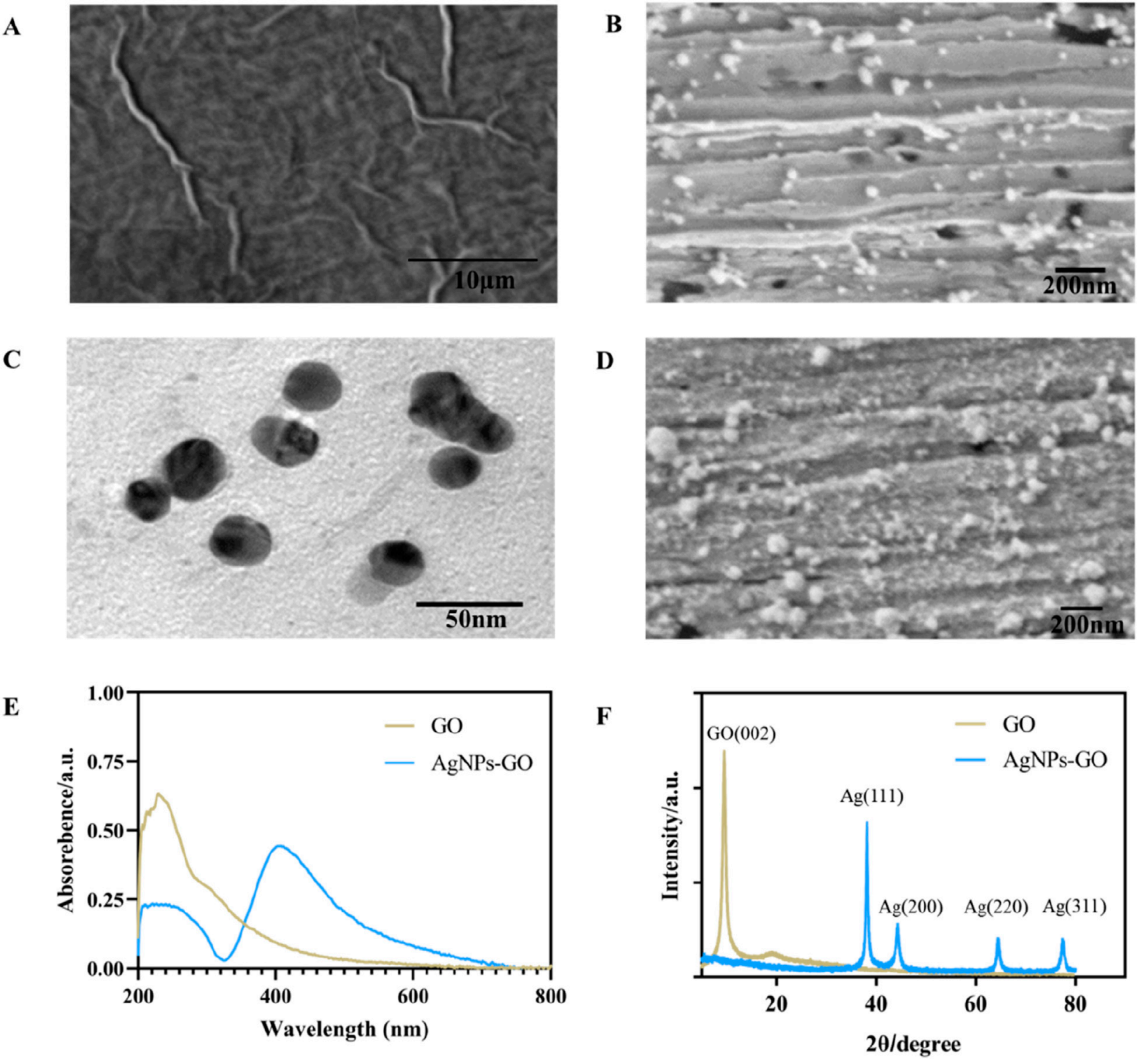
**(A)** The SEM image of GO. **(B)** The SEM image of AgNPs-GO nanocomposites. **(C)** The TEM image of AgNPs-GO nanocomposites. **(D)** The SEM image of AuNPS/AgNPs-GO nanocomposites. **(E)** The UV-Vis spectra of GO and AgNPs-GO nanocomposites. **(F)** The XRD patterns of GO and AgNPs-GO nanocomposites.

SEM was applied to observe the morphology of nanomaterials. As can be seen from Figure 2A, GO presented a thin, spongy structure with folds. In comparison, Figure 2B showed the successful *in-situ* reduction of a large number of AgNPs to the surface of the GO sheet structure, Supplementary Figures S1A–C (SEM-EDX mappings) and Supplementary Figure S1D (SEM-EDX spectra) showed that the nanocomposites on the gold electrode mainly consisted of C, O, Ag and Au, suggesting the successful preparation of the AgNPs-GO nanocomplex. Meanwhile, TEM images in Figure 2C showed that AgNPs had a typical crystal morphology, with an average diameter of about 26 nm. Figure 2D showed that AuNPs were modified onto AgNPs-GO nanocomposites by potentiostatic electrodeposition. On the UV characterization of GO and AgNPs-GO, as shown in Figure 2E, the characteristic absorption peak of GO was 230 nm (Musa et al., 2023). In addition to the absorption peak at 230 nm, the characteristic absorption peak of Ag also appeared at 410 nm (Lu et al., 2017), which also proved that the AgNPs-GO nanocomposites was successfully synthesized. XRD diffraction diagram in Figure 2F showed the strong diffraction peak with 2θ angles around 10° was classified as the (002) crystal plane of GO (Kaur et al., 2023). In AgNPs-GO nanocomposites, the diffraction peaks around 38°, 44°, 65° and 78° corresponded to the (111), (200), (220) and (311) crystal planes of silver, respectively (Adamopoulos et al., 2023). It was worth noting that the diffraction peak of GO in the AgNPs-GO nanocomposites disappeared, indicating that the graphene oxide was stripped (Parsaee et al., 2018).

### 3.2 Electrochemical characterization of the aptasensor

**FIGURE 3:**
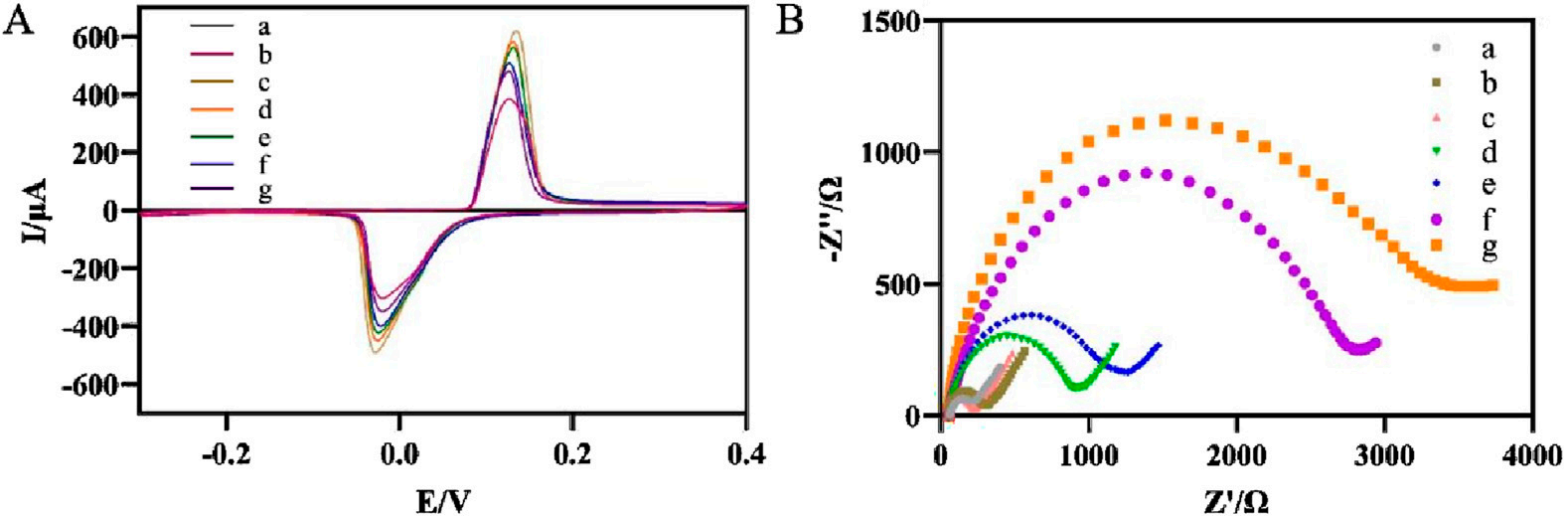
**(A)** Recorded CVs of the modified electrode after each immobilization. (a) Au electrode, (b) AgNPs-GO/Au, (c) AuNPs/AgNPs-GO/Au, (d) SA/AuNPs/AgNPs-GO/Au, (e) Apt/SA/AuNPs/AgNPs-GO/Au, (f) BSA/Apt/SA/AuNPs/AgNPs-GO/Au, (g) Hp/BSA/Apt/SA/AuNPs/AgNPs-GO/Au. **(B)** Nyquist curves for different steps of the modified electrode. a-g were the same as **(A)**.

Figure 3A showed the response curves of different modified electrodes during CV test in PBS (0.1 M, pH 7.0). The gold electrode did not show a redox response in PBS (curve a) due to the absence of oxidation-reduction electronic intermediaries. When AgNPs-GO nanocomposites were dropped onto the gold electrode, the modified electrode showed a good pair of oxidation-reduction current responses due to the excellent oxidation-reduction characteristics of the AgNPs (Walgama and Raj, 2023) (curve b), indicating that the AgNPs-GO nanocomposites had good oxidation-reduction activity. After further electrodeposition of AuNPs on the modified electrode surface, a significantly enhanced current signal was observed (curve c). This was because AuNPs had excellent conductivity, which could promote the transmission of electrons. After the streptavidin, which was electrically inert, was immobilized on the electrode surface, the current signal decreased (curve d). When biotin-modified aptamer was immobilized on the electrode surface, the current signal decreased again, indicating that the biotin-modified aptamer blocked the channel for electron hopping (curve e). After non-conductive BSA was used to block the non-specific binding sites on the electrode, the current signal further decreased (curve f). After binding to *H. pylori*, the current signal decreased again (curve g), which was attributed to the formation of aptamer-*H. pylori* complexes blocking the electron transfer.

EIS was used to further characterize the stepwise modification of the aptasensor (Figure 3B). In contrast to the impedance of the bare gold electrode (curve a), the impedance values of the AgNPs-GO modified electrode (curve b) and the subsequently modified electrode with AuNPs (curve c) decreased in turn, indicating that AgNPs-GO and AuNPs had good conductivity, thus facilitating the electrons transfer. The modification of streptavidin (curve d) and biotin-modified aptamer (curve e) increased the values of impedance in turn, suggesting that both streptavidin and biotin-modified aptamer impeded the transport of electrons. The values of impedance further increased after BSA (curve f) and *H. pylori* (curve g) were dropped on the modified electrode due to the poor electrical conductivity of BSA and the bacteria (Liu et al., 2025). The results of EIS were consistent with those of CV, showing that we successfully prepared the electrochemical aptasensor.

### 3.3 Optimization of experimental conditions

**FIGURE 4:**
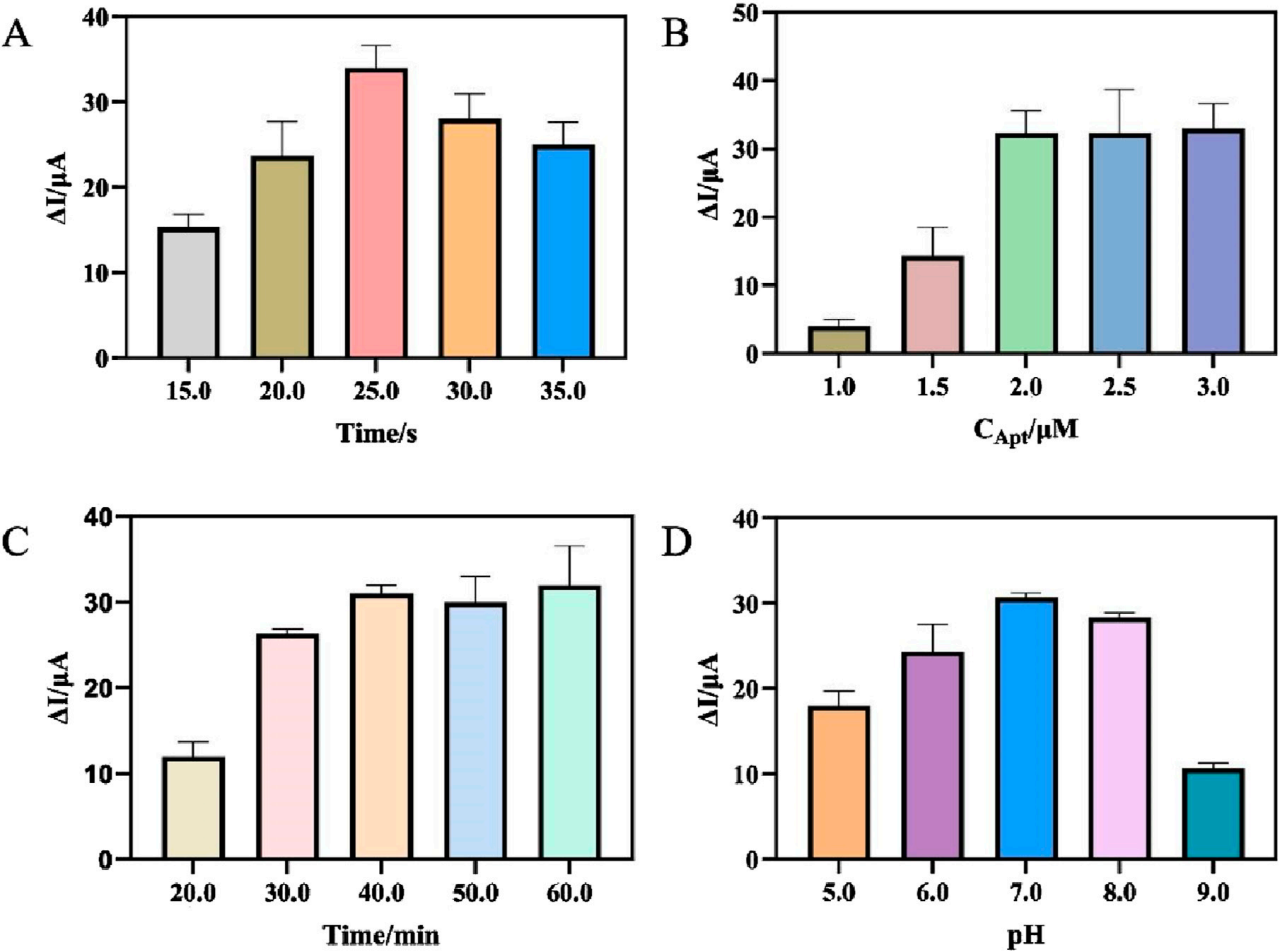
**(A)** Optimization of the electrodeposition time of AuNPs. **(B)** Optimization of the concentration of Apt. **(C)** Optimization of the incubation time of *Helicobacter pylori*. **(D)** Optimization of the pH of the detection solution. (error bar = SD, n = 3)

Optimizing experimental conditions aimed to improve the analytical performance of the aptasensor and enhance its sensitivity, including the electrodeposition time of AuNPs, the concentration of aptamer, the incubation time of *H. pylori* and the pH of the detection solution. With the increase of electrodeposition time, a large number of AuNPs were modified onto the electrode surface, effectively fixing SA and possibly preventing the loss of oxidation-reduction probe AgNPs-GO. However, excessive electrodeposition time may result in an excessively thick coating of AuNPs which blocked the channels for electron hopping and hindered the improvement of electrochemical signal (Wu et al., 2017). Figure 4A showed that the change of current (ΔI) increased with the extension of electrodeposition time and reached its maximum value at 25s. Therefore, the optimal electrodeposition time for the aptasensor was chosen as 25s. Next, the concentration of aptamer fixed on the electrode was optimized. The results were shown in Figure 4B. As the concentration of aptamer increased, the ΔI initially increased and then stabilized. When the concentration of aptamer was greater than 2 μM, the ΔI was basically unchanged. Therefore, in this experiment, the aptamer concentration of 2 μM was adopted. The ability to effectively capture the target *H. pylori* was also an important factor affecting the sensor. Therefore, the incubation time of specific reaction between aptamer and *H. pylori* was optimized. As can be seen from Figure 4C, with the increase of incubation time, the ΔI gradually increased, and the ΔI basically remained unchanged after 40 min. Therefore, 40 min was selected as the optimal incubation time. Finally, the electrochemical response in detection solutions with different pH was examined. As shown in Figure 4D, too high (above 7.0) or too low (below 7.0) of the pH were not favorable for the aptasensor to respond to *H. pylori*. Therefore, PBS with a pH of 7.0 was used as the detection solution.

### 3.4 Analytical performance of the aptasensor for *Helicobacter pylori*

**FIGURE 5:**
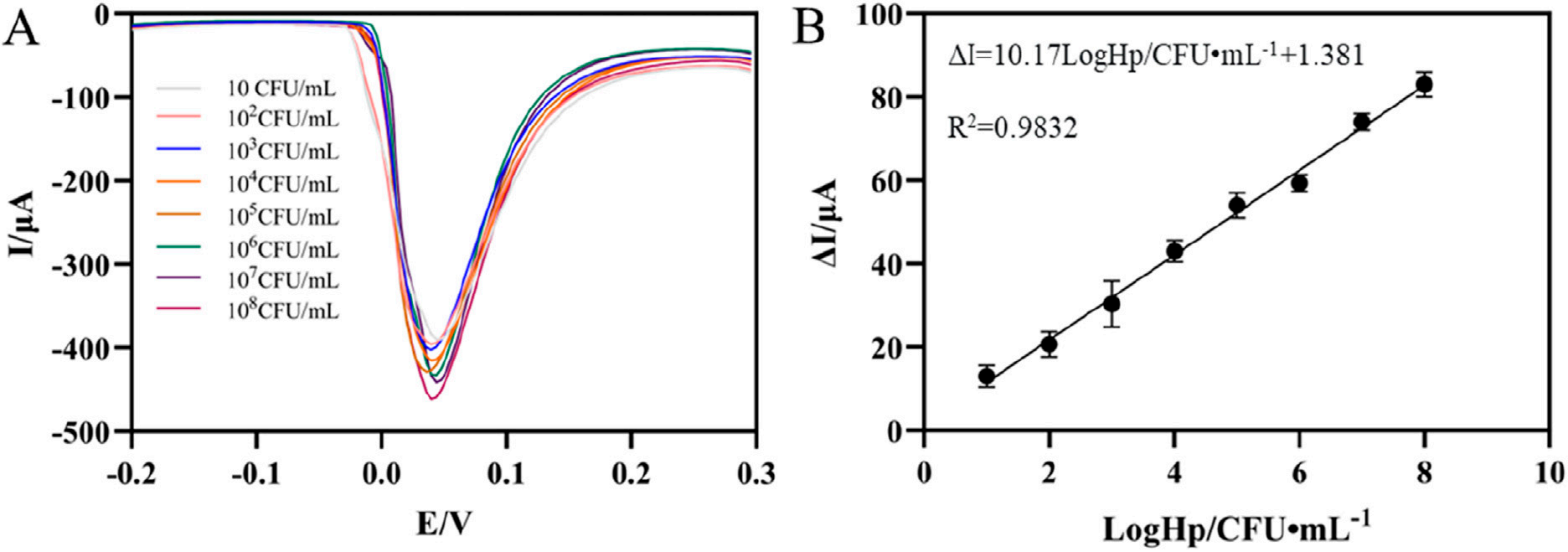
**(A)** DPV of the aptaensor incubated with different concentrations of *Helicobacter pylori*. **(B)** Calibration plots of ΔI versus the logarithm of *Helicobacter pylori* concentrations (error bar = SD, n = 3)

Under optimal experimental conditions, DPV tests were performed in PBS (pH7.0). As can be seen from Figure 5A, DPV signal decreases correspondingly with the increase of *H. pylori* concentration. This was because the increase of *H. pylori* caused more aptamer-*H. pylori* complex to be attached to the electrode surface, thereby impeding the transfer of electrons on the electrode surface, resulting in a decrease in the electrochemical signal. It can be seen from Figure 5B that in the concentration range of 10^1^∼10^8^ CFU mL^−1^, the change of the response current had a good linear relationship with the logarithmic value of the *H. pylori* concentration. The corresponding linear equation was ΔI = 10.17LogHp/CFU mL^−1^+1.381 (R^2^ = 0.9832). The detection limit was 3 CFU mL^−1^ (S/N = 3), reflecting the high sensitivity of the sensor. Compared with the previously reported detection methods of *H. pylori*, the results in Table 1 showed certain advantages in the linear range and LOD (Musa et al., 2023; Zou et al., 2022; Sarabaegi et al., 2022; Ning et al., 2023). This came down to three factors. First, the large surface area and high conductivity of AgNPs-GO could increase the current signal. Secondly, AuNPs had high electrical conductivity and good biocompatibility, which increased the current signal and the streptavidin retention. Thirdly, through the specific binding of streptavidin and biotin, the adhesive load of aptamer on the electrode surface was increased, thus improving the sensitivity of the aptasensor. Furthermore, although the detection limits of some sensors were superior to those in this study (Saber Mirzaei et al., 2024), AgNPs was used as the redox probe in this work, thereby avoiding the addition of additional redox probes and having the advantage of simplifying the preparation process of aptamer sensors.

**TABLE 1:** Comparison of different analytical methods for *Helicobacter pylori* detection.

Methods	Linear range	Detection limit	Samples	Ref.
Electrochemical aptaensor	10^2^∼10^7^ CFU mL^−1^	33 CFU mL^−1^	Blood serum	Chen et al. (2023)
Fluorescent aptasensor	20–1000 CFU mL^−1^	5 CFU mL^−1^	Stool	Walgama and Raj (2023)
Electrochemical aptaensor	10^1^∼10^7^ CFU mL^−1^	1 CFU mL^−1^	Blood serum	Liu et al. (2025)
LSPR aptasensor	10^2^∼10^8^ CFU mL^−1^	45 CFU mL^−1^	Water	Wu et al. (2017)
Electrochemical aptaensor	10^1^∼10^8^ CFU mL^−1^	3 CFU mL^−1^	Blood serum/stool	This work

### 3.5 Reproducibility, stability and selectivity of the aptasensor

**FIGURE 6:**
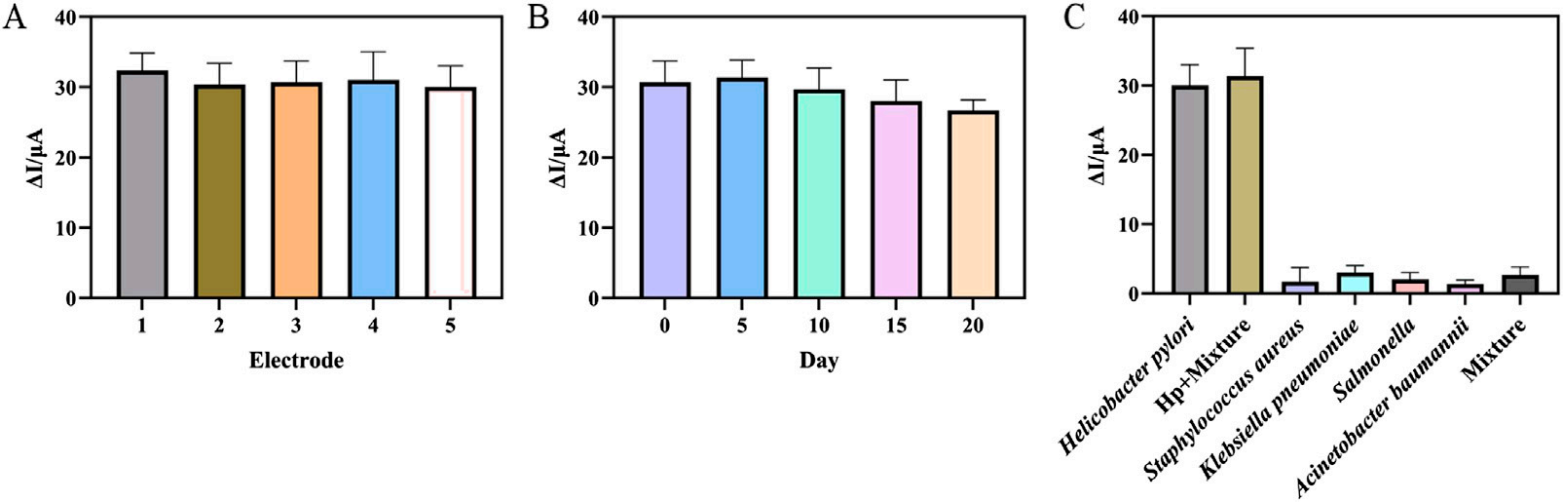
**(A)** Five different aptasensor treated in the same way at 10^3^ CFU mL^−1^ *Helicobacter pylori*. **(B)** The ΔI towards different storage time at 10^3^ CFU mL^−1^ *Helicobacter pylori*. **(C)** The ΔI towards *Helicobacter pylori*, *Helicobacter pylori* + Mixture (*Staphylococcus aureus*, *Klebsiella pneumoniae*, *Salmonella enterica* and *Acinetobacter baumannii*), *Staphylococcus aureus*, *Klebsiella pneumoniae*, *Salmonella enterica* and *Acinetobacter baumannii* and mixture. (error bar = SD, n = 3)

Reproducibility, stability and selectivity were assessed to examine the electrochemical performance of the prepared aptasensor. Firstly, five aptasensors were used to detect *H. pylori* with the same concentration to investigate the reproducibility. The results were shown in Figure 6A, a relative standard deviation of 2.92% indicated that the reproducibility of the aptasensor was acceptable. Secondly, in order to investigate the stability of the aptasensor, the prepared aptasensor was stored at 4°C and tested every 5 days. The results in Figure 6B showed that after 15 days, the response current of the aptasensor was 91.32% of the initial value, and after 20 days, the ΔI was 86.95% of the initial value. The results indicated that the aptasensor had acceptable stability in the detection of *H. pylori*. Finally, four interfering bacteria were incubated on the aptasensor instead of *H. pylori* to investigate the selectivity of the aptasensor. As shown in Figure 6C, the sensor incubated with *H. pylori* showed the largest corresponding current change. When only interfering bacteria were incubated, the sensor showed almost no current change. The DPV peak current change for the mixture of the *H. pylori* and interfering substance was almost the same as the electrical signal changes when only *H. pylori* was present. This showed that the aptasensor had good selectivity, which was due to the specific binding of aptamer and *H. pylori*.

### 3.6 Application of aptasensor in real samples

We used the standard addition method to evaluate the reliability and practicality of the aptasensor. First, different concentrations of *H. pylori* were added to blood serum and stool samples to prepare the samples. The samples were obtained from C-UBT-negative patients, proving that the sample did not contain *H. pylori*. Blood serum was diluted with PBS solution at a ratio of 1:2, and stool sample (1 g) was mixed with 200 μL of PBS solution to remove the matrix effect (Supplementary Figure S2). *H. pylori* was added to the samples, with final concentrations including 10^3^ CFU mL^−1^, 10^5^ CFU mL^−1^, and 10^7^ CFU mL^−1^. DPV signals was showed in Supplementary Figure S3, and as can be seen in Table 2, the recovery rates of *H. pylori* in blood serum were 93.49%–102.56%, with relative standard deviations of 5.66%–7.99%. The recovery rates of *H. pylori* in stool samples were 97.08%–106.59%, with relative standard deviations of 6.65%–8.76%. These results indicated that the aptasensor had high accuracy and had the potential for practical application in sample detection.

**TABLE 2:** Detection of *Helicobacter pylori* in blood serum and stool samples (n = 3).

Sample	Added value (CFU mL^−1^)	Detected (CFU mL^−1^)	Recovery (%)	RSD (%)
blood serum	10^3^	0.97 × 10^3^	97.33	5.66
	10^5^	1.03 × 10^5^	102.56	7.99
	10^7^	0.93 × 10^7^	93.49	6.99
stool	10^3^	1.07 × 10^3^	106.59	6.65
	10^5^	1.01 × 10^5^	101.06	8.76
	10^7^	0.97 × 10^7^	97.08	6.84

## 4 Conclusion

In this work, we successfully prepared a high-sensitivity and label-free electrochemical aptasensor for the detection of *H. pylori* using AuNPs/AgNPs-GO nanocomposites and streptavidin. AgNPs-GO nanocomposites not only had large surface area and high conductivity, but also provided a good pair of redox peaks. AuNPs had good electrical conductivity, which could amplify electrochemical signals, and its strong biocompatibility firmly supported streptavidin on the modified electrode. The biotin-modified aptamer could be attached to the modified electrode surface by the specific adsorption of streptavidin and biotin, thus improving the sensitivity of the aptasensor. In addition, the aptasensor did not need to introduce additional redox electron mediators, thus simplifying electrode preparation and reducing costs. Therefore, the aptasensor showed a wide linear range, low detection limit, good selectivity and stability, which provided a choice for the establishment of an economical and effective vector-free electrochemical aptasensor.

## Data Availability

The original contributions presented in the study are included in the article/Supplementary Material, further inquiries can be directed to the corresponding authors.
