# Supplementary material for: An electrochemical aptasensor for detection of Helicobacter pylori based on AuNPs and AgNPs-GO nanoparticles
Source: Front Bioeng Biotechnol. 2025 Jul 9;13:1619336. doi: 10.3389/fbioe.2025.1619336 (PMC12283743; doi:10.3389/fbioe.2025.1619336)
Supplement: Supplementary file 1 [file DataSheet1.docx]

**Supporting Information**

**An Electrochemical Aptasensor For Detection of *Helicobacter pylori* Based on AuNPs and AgNPs-GO Nanoparticles**

Xiaojuan You^1,2,3^, Mingyi Shao^1,2*^, Huadong Wang^4^, Rui Zhu^3^, Xinwei Liu^3^, Lei Dong^1^, Yuesheng Gong^1,*^, Yongwei Li^1,3*^

^1^Zhengzhou Anorectal Hospital, Zhengzhou 450004, China

^2^The First Affiliated Hospital of Henan University of Chinese Medicine, Zhengzhou 450000, China

^3^The Second Clinical Medical College of Henan University of Chinese Medicine, Zhengzhou, 450002, China

^4^Henan Center For Drug Evaluation and Inspection, Zhengzhou 450008, China

*Corresponding author.

E-mail address: shmy1016@163.com (Mingyi Shao), kyzhengzhougc@163.com (Yuesheng Gong), lyw@hactcm.edu.cn (Yongwei Li)

**This part includes:**

**1. Synthesis of *H. pylori* aptamers**

The synthesis of *H. pylori* aptamers was based on Reference 23. The detailed steps were as follows. Nucleic acid aptamers were screened using *H. pylori* surface recombinant antigens as targets. Trypsin was used for separating aptamers that were bound to proteins. Following nine rounds of screening, *H. pylori* aptamer with the strongest binding ability to the *H. pylori* surface recombinant antigen was chosen. After optimization of the binding conditions, specificity tests for *H. pylori* aptamer were conducted using *Escherichia coli*, *Staphylococcus aureus*, *Vibrioanguillarum*, and *H pylori.* The data indicated that the *H. pylori* aptamer had an equilibrium dissociation constant (Kd) of 26 nmol/L to the target protein. This aptamer was capable of exclusively detecting *H. pylori*, without displaying any specificity for other bacteria.

In order to modify the aptamer onto the electrode, 3' of the *H. pylori* aptamer combined with biotin. Through the specific mutual recognition of streptavidin and biotin, the aptamer was fixed to the electrode, and subsequent detection of *H. pylori* was carried out.

**2. SEM-EDX mappings of AgNPs-GO nanocomposites**


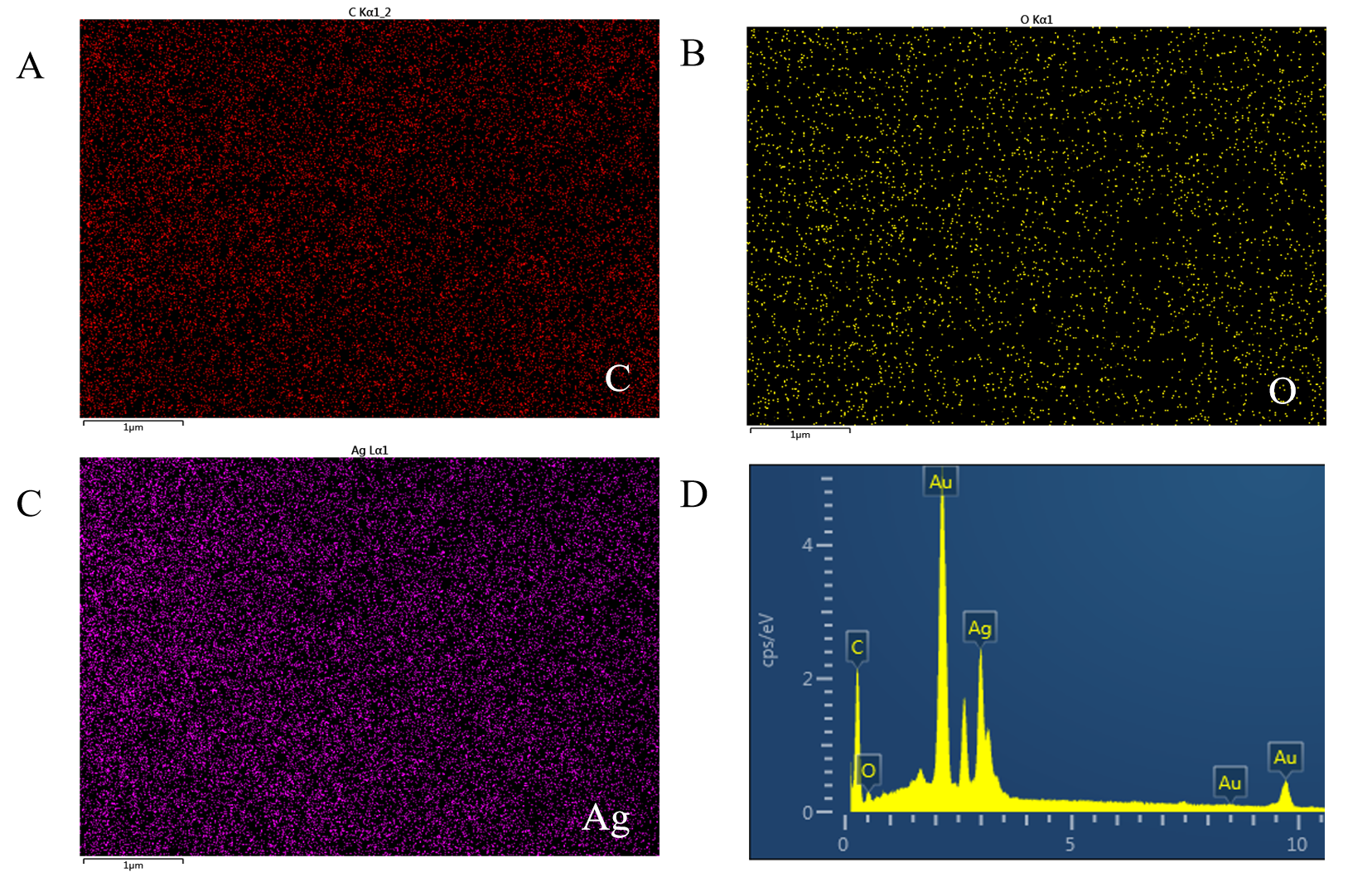


Fig. S1. SEM-EDX mappings of C (A), O (B), Ag (C) and SEM-EDX spectra (D).

**3. Optimization of matrix effect**

The method of diluting the reals samples was adopted to remove the influence of fouling on the electrode. The blood samples were diluted at different multiples, as shown in Figure S1 (A). When the blood samples were diluted to more than 2 times, the DPV standard curve was similar to that of PBS, indicating that diluting the blood samples to more than 2 times could basically eliminate the influence of the matrix effect. The matrix effect of stool samples was determined by the same method, and the results were shown in Figure S1 (B). When stool sample (1 g) was mixed with 200 μL of PBS solution, the matrix effect of the samples could basically be eliminated.


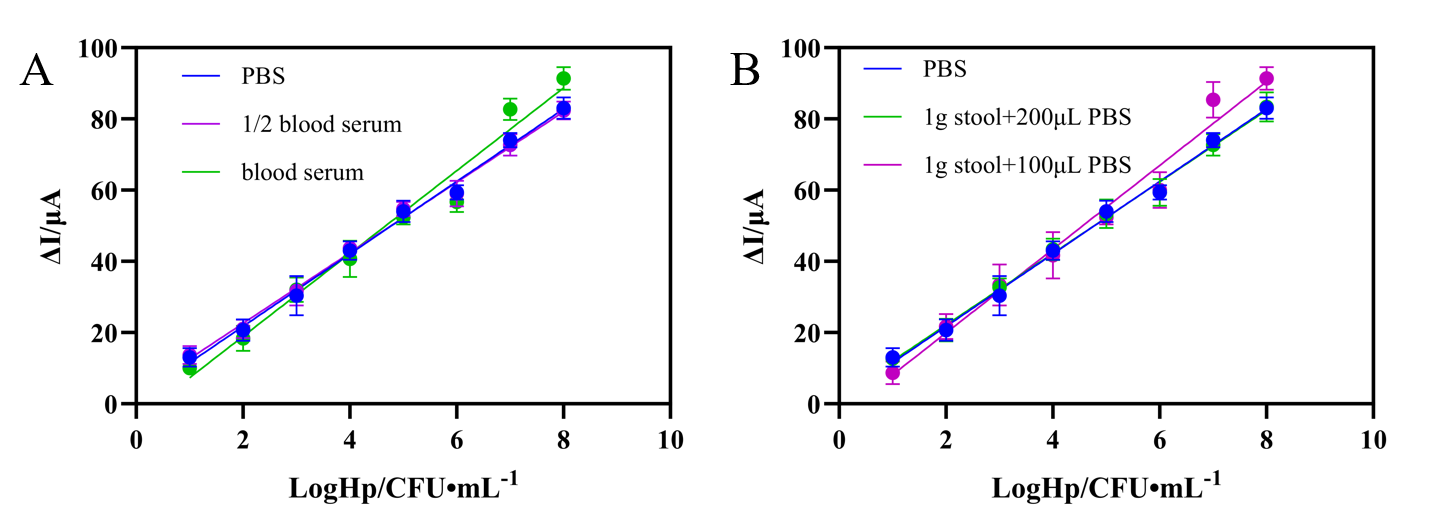


Fig. S2 (A) Optimization of matrix effects in blood serum samples. (B) Optimization of matrix effects in stool samples

**4. DPV signals of blood serum and stool samples**


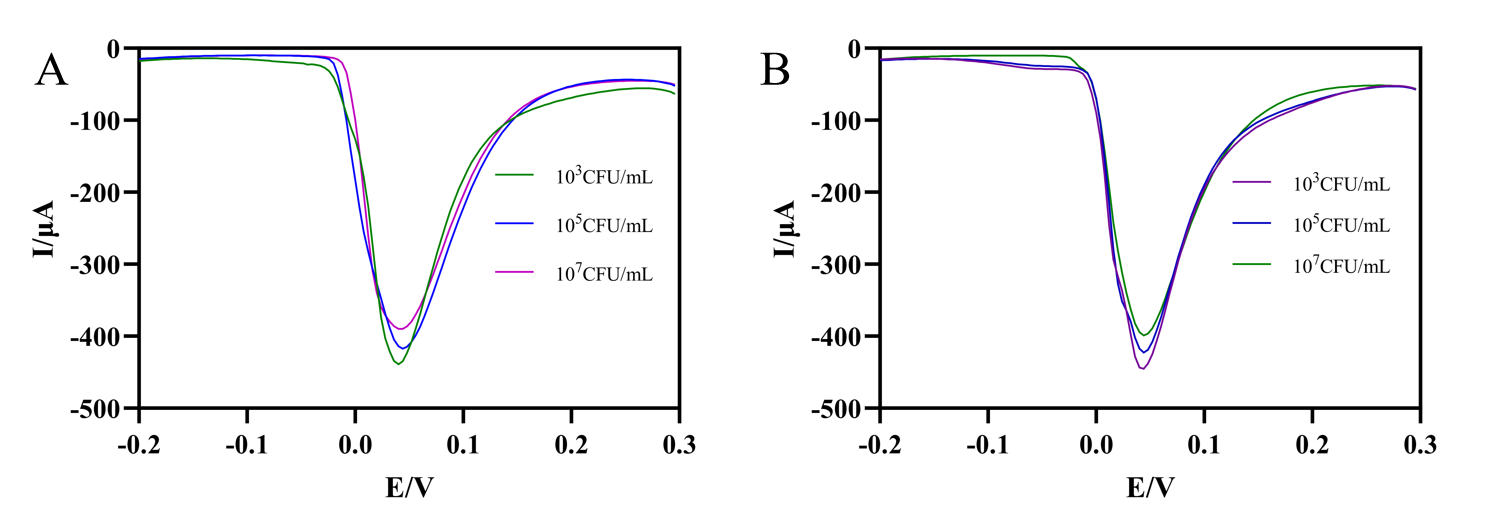


Fig. S3 (A) DPV signals of blood serum samples. (B) DPV signals of stool samples.
